# Preparation and study of characteristics of LiCoO_2_/Fe_3_O_4_/Li_2_B_2_O_4_ nanocomposites as ideal active materials for electrochemical hydrogen storage

**DOI:** 10.1039/d1ra02453a

**Published:** 2021-07-05

**Authors:** Mahdi Ranjeh, Omid Amiri, Masoud Salavati-Niasari, Mehdi Shabani-Nooshabadi

**Affiliations:** Institute of Nano Science and Nano Technology, University of Kashan Kashan P. O. Box 87317-51167 I. R. Iran salavati@kashanu.ac.ir +98 315 5913201 +98 315 5912383; Faculty of Chemistry, Razi University Kermanshah 6714414971 Iran; Department of Chemistry, College of Science, University of Raparin Rania Kurdistan Region Iraq

## Abstract

The nanocomposites of LiCoO_2_/Fe_3_O_4_/Li_2_B_2_O_4_ were designed by the Pechini route using different fuels for the optimization of their morphology and structure. Compared to other fuels, citric acid can act as both an ideal fuel and a capping agent. The ratio of the EG : H_2_O mixture is another parameter, which was studied in terms of its effects on the structural characterization. The optimized sample with a rod shape was selected to compare with the bulk sample through electrochemical hydrogen storage capacity. The discharge capacity for rod-shaped nanocomposites measured was 1284 mA h g^−1^. However, the discharge capacity for the bulk morphology was calculated to be about 694 mA h g^−1^. The magnetic, electrochemical and structural analyses were performed to investigate the properties of LiCoO_2_/Fe_3_O_4_/Li_2_B_2_O_4_ nanocomposites.

## Abstract 2

To the best of our knowledge, for the first time, the effects of LiCoO_2_/Fe_3_O_4_/Li_2_B_2_O_4_ nanocomposites as a favorable hydrogen capacitor were investigated to enhance hydrogen storage performance.

## Graphical abstract

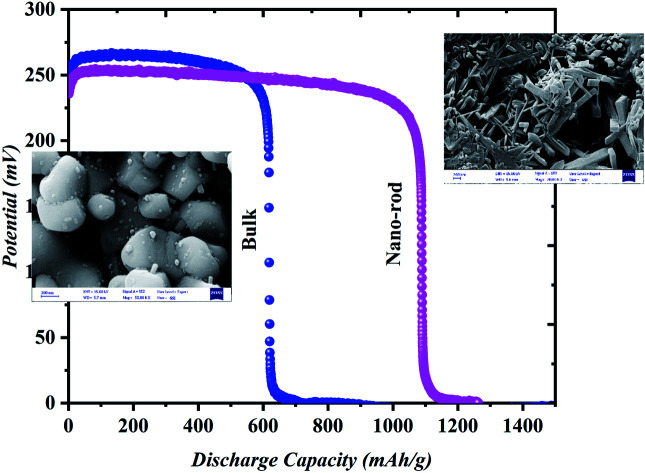

## 1. Introduction

At present, the usage of fossil fuels has been restricted due to the release of pollutants from their combustion and the defective origins. In order to meet the energy demands, researchers are designing prevalent and up-to-date replacement sources for energy storage. Electrochemical hydrogen sorption is a potential technique for energy storage in the nano-scale compounds in terms of reproducibility, safety and clean sources because of their availability, supreme proficiency, simple technique and low pollution. Designing novel active materials with improved efficiency, amplified structure or compound, and sustainable consumption is a crucial target in energy storage fields.^1–3^

The structural stability, structural chemistry, mechanical attributes, and physical properties are remarkable factors for the selection of energy storage materials. The selection of suitable active materials and the design of an improved structure are important tasks for achieving high performance in electrochemical energy storage. Therefore, metal oxide-supported materials are key options to design potential active materials for electrodes utilized in energy storage.^4–6^

Nano-scale structures with different compositions are synthesized for the energy storage application *via* chemical procedures, which are simple and afford high yield products. These chemical methods include ultrasonic-based precipitation, microwave-based precipitation, fuel-assisted sol–gel method, solvothermal and thermal treatment routes.^7–10^ The metal oxide materials applied in the energy storage fields are comprehensive such as borate, vanadate, spinels, and perovskites. The central metals in these structures usually are cobalt, iron, nickel, magnesium, zinc, *etc.* The important factor for the selection of the central metal is the ability to change the oxidation number in the redox process during electrochemical charge and discharge.^11–15^

In the recent study, the ternary nanocomposites of LiCoO_2_/Fe_3_O_4_/Li_2_B_2_O_4_ were designed by the one-step Pechini procedure by changing the operational synthesis parameters to achieve an ideal morphology. The ideal morphology of rod-shape in the nano-scale was achieved using citric acid and compared with the bulk structures in terms of electrochemical hydrogen sorption properties. The designed rod-shaped LiCoO_2_/Fe_3_O_4_/Li_2_B_2_O_4_ nanocomposites show a discharge capacity of 1284 mA h g^−1^ after 15 cycles. Compared to the bulk structures of these nanocomposites with a capacity of 694 mA h g^−1^, the results confirm the effect of decrease in the size of the sample for hydrogen storage performance of LiCoO_2_/Fe_3_O_4_/Li_2_B_2_O_4_ nanocomposites. Overall, this is the first research to acquire suitable hydrogen capacitor materials synthesized *via* an easy Pechini method in order to achieve an economic, simple and quick technique.

## 2. Experiments

Nanocomposites of LiCoO_2_/Fe_3_O_4_/Li_2_B_2_O_4_ were successfully designed through the Pechini route in the presence of diverse fuels. In the first step, Co(NO_3_)_2_·6H_2_O, Fe(NO_3_)_3_·9H_2_O, Li_2_SO_4_·H_2_O and H_3_BO_3_ were dissolved in the specified mixing solvent of ethylene glycol and water. The mixture of the solvent was prepared by different ratios of EG and H_2_O, as summarized in Table 1. Then, the chosen fuel materials were added to the above-mentioned mixture. Four types of structures such as EDTA, phthalic acid, citric acid and tartaric acid could play the dual role in the Pechini reaction as a chelating agent and as a fuel to control the morphology and perform the synthesis reaction. Ethylene diamine was applied in order to control the pH of the reaction at 10. The mixture was heated at 80 °C to form a gel. The obtained gel was dried for 4 h in an oven at 100 °C. Eventually, the dried gel was heat-treated for 4 h at 700 °C to obtain the products.

**Table 1:** Summary of the synthesized conditions for Pechini-designed nanocomposites

Sample no.	Fuel	EG : H_2_O
S1	EDTA	1 : 1
S2	Phthalic acid	1 : 1
S3	Citric acid	1 : 1
S4	Tartaric acid	1 : 1
S5	Citric acid	2 : 1
S6	Citric acid	3 : 1
S7	Citric acid	4 : 1

In order to investigate the hydrogen sorption capacity of the samples, the electrochemical chronopotentiometry technique was utilized in the assembled cell using Pt (counter electrode), Ag/AgCl (reference electrode) and working electrodes in the electrolyte medium of KOH (2 M). The applied current density is constant 1 mA. Therefore, the potentials of the working electrode *versus* reference electrode were recorded by applying constant current between the working and counter electrodes. The working electrode was prepared by coating a thin layer of fabricated nanocomposites on the copper substrate, which dried at 60 °C to form a uniform layer on the copper foam.

## 3. Characterization details

**Fig. 1:**
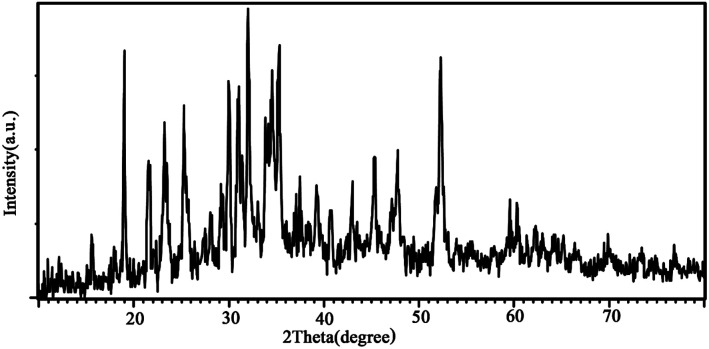
XRD diffractogram of sample 3 (synthesized by citric acid).

**Fig. 2:**
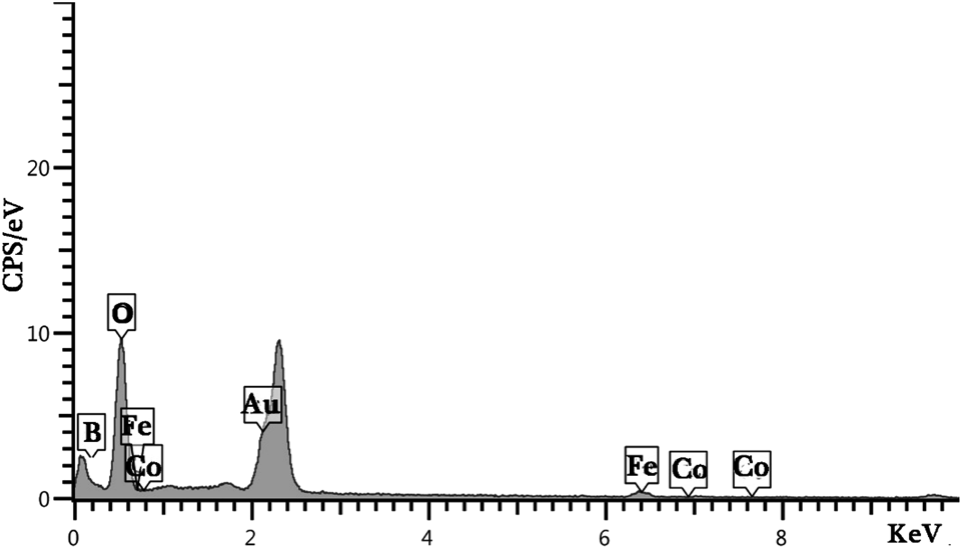
EDS result for sample 3 (synthesized by citric acid).

In order to certify the phase purity of the designed nanocomposites, the XRD diffractograms were recorded *via* the X-ray diffraction technique. As shown in Fig. 1, the three components existed in the texture of composites. LiCoO_2_ (JCPDS 16-0427), Fe_3_O_4_ (JCPDS 89-0951) and Li_2_B_2_O_4_ (JCPDS 11-0407) items can match with the displayed diffracted peaks. The sharp diffracted peaks present a highly crystallized structure for designed nanocomposites. The EDS result for sample 3 is presented in Fig. 2. The result of the energy dispersive spectroscopy confirms that the nanocomposites are composed of B, Co, Fe and O elements (Fig. 2). It is well-known that the EDS pattern reveals the high purity of the product.

**Fig. 3:**
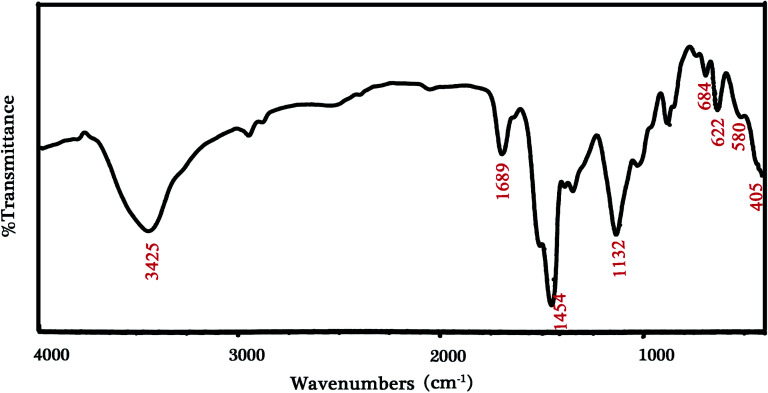
FT-IR profile for sample 3 (synthesized by citric acid).

The achieved profile for sample 3 is presented in Fig. 3. The situated peaks at 405, 684 and 1132 cm^−1^ related to Li_2_B_2_O_4_ structures in terms of vibrating Li–O, bending B–O–B and stretching B–O bonds, respectively. The stretching mode of the [CoO_6_] octahedron is supported by the peak located at 622 cm^−1^, which corresponds to the LiCoO_2_ structure. The observed weak peak at 580 cm^−1^ can be correlated to the Fe_3_O_4_ structure. Also, the remaining molecules of water and carboxylic acid create the bonds of O–H and C–O at 3425, 1689 and 1454 cm^−1^.^11,16,17^

**Fig. 4:**
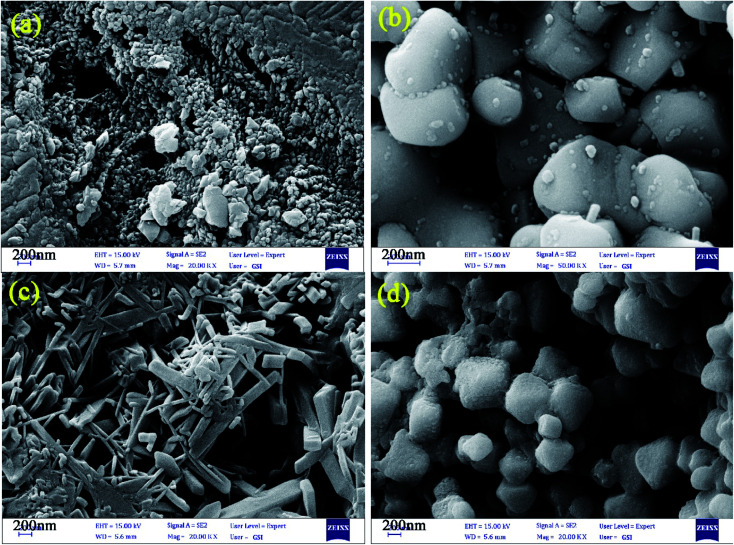
FE-SEM images of nanocomposites designed by using (a) EDTA, (b) phthalic acid, (c) citric acid, and (d) tartaric acid [sample 1–4].

**Fig. 5:**
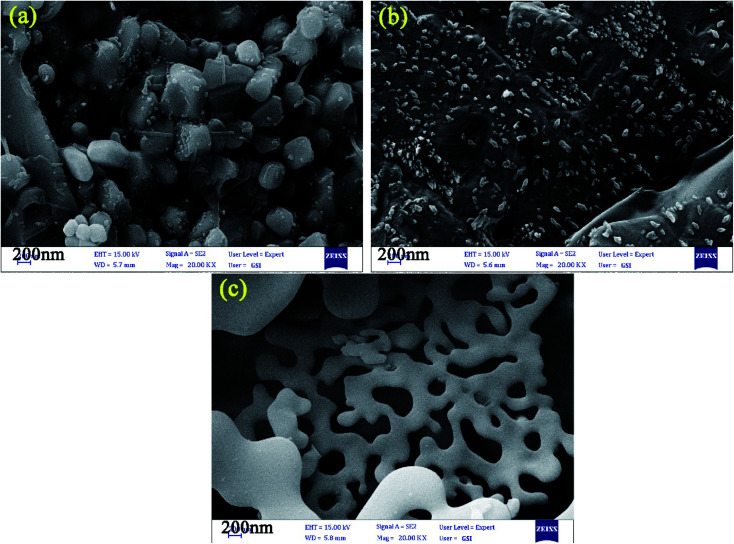
FE-SEM images of the designed nanocomposites by citric acid in the medium of EG : H_2_O by different ratios (a) 2 : 1, (b) 3 : 1 and (c) 4 : 1.

**Fig. 6:**
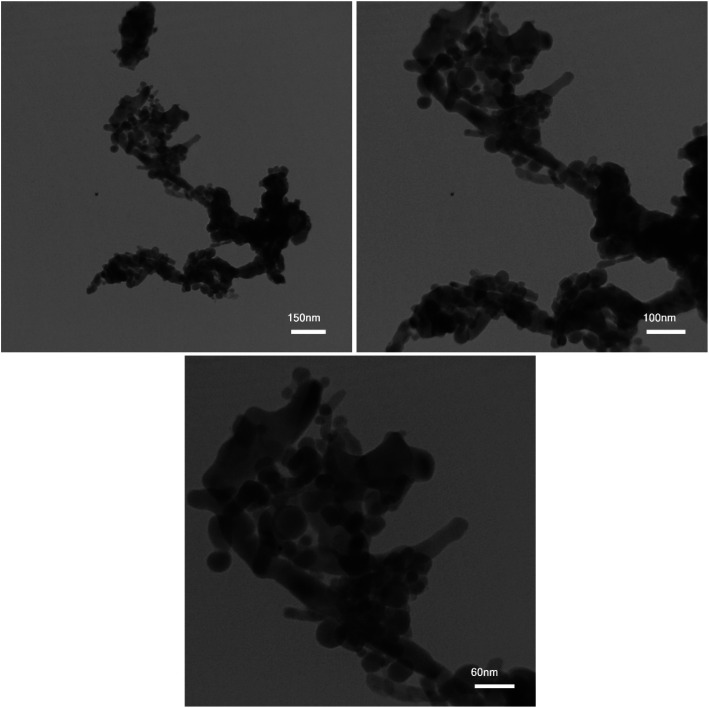
TEM images for the synthesized nanocomposites by citric acid (sample 3).

The control of the morphology is a significant issue in the design of nanomaterials, which are applied for energy storage applications. In this study, the effects of diverse fuels on the morphology of the as-synthesized nanocomposites were compared as the first parameter due to the chelating agent role of chosen fuel structures. Fig. 4a–d display the FE-SEM images of the designed nanocomposites in the presence of EDTA, phthalic acid, citric acid, and tartaric acid, respectively. The EDTA-assisted Pechini-synthesized nanocomposites having agglomerated structures are shown in Fig. 4a. The FE-SEM images of the as-fabricated nanocomposites using phthalic acid and tartaric acid (Fig. 4b and d) show the formation of large structures in microscale. As shown in Fig. 4c, the nanocomposites fabricated using citric acid possessed rod-shaped structures in nano-scale. The second parameter for controlling the morphology in this study is the ratio of EG : H_2_O in the reaction medium owing to the role of EG as a gelling agent. Fig. 5a–d represents the ratios of 2 : 1, 3 : 1 and 4 : 1 of EG : H_2_O, respectively. FE-SEM images for samples 5 and 6 exhibit large and agglomerated structures (Fig. 5a and b). However, sample 7 (Fig. 5c) prepared in the mixture of EG : H_2_O (4 : 1) shows porous structures. Comparing the FE-SEM results, it is found that the designed nanocomposites in the presence of citric acid and the ratio of 1 : 1 for EG : H_2_O present an ideal morphology and size, which was chosen as the optimum sample (sample 3). To further analyze the morphology and shape of the optimized sample, the TEM images were obtained for sample 3, as shown in Fig. 6. The results of the obtained TEM images confirm the morphology of sample 3.

**Fig. 7:**
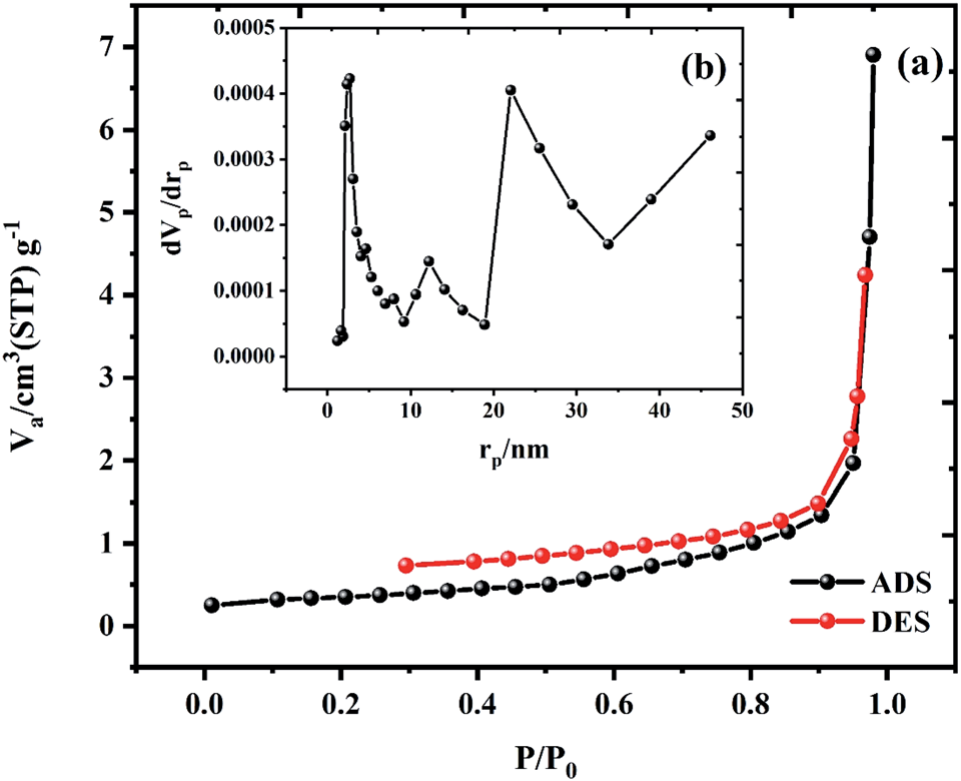
(a) BET and (b) BJH plot for sample 3.

The porosity and surface characteristics were determined using the BET method for sample 3, which is presented in Fig. 7. The BET method was also applied to assess the surface area and pore volume of the designed nanocomposites with rod-shaped structures, as shown in the nitrogen adsorption isotherms measured at 77 K. The specific surface area, pore volume, and average pore size for sample 3 is 1.1896 m^2^ g^−1^, 0.010677 cm^3^ g^−1^, and 35.902 nm, respectively. The pore size distribution of the nanocomposites is displayed in Fig. 7b. Finally, BET results affirm that the surface area and porosity are important issues for the facile ion transportation to increase the electrochemical performance of the active materials in the electrode matrix.^18^

**Fig. 8:**
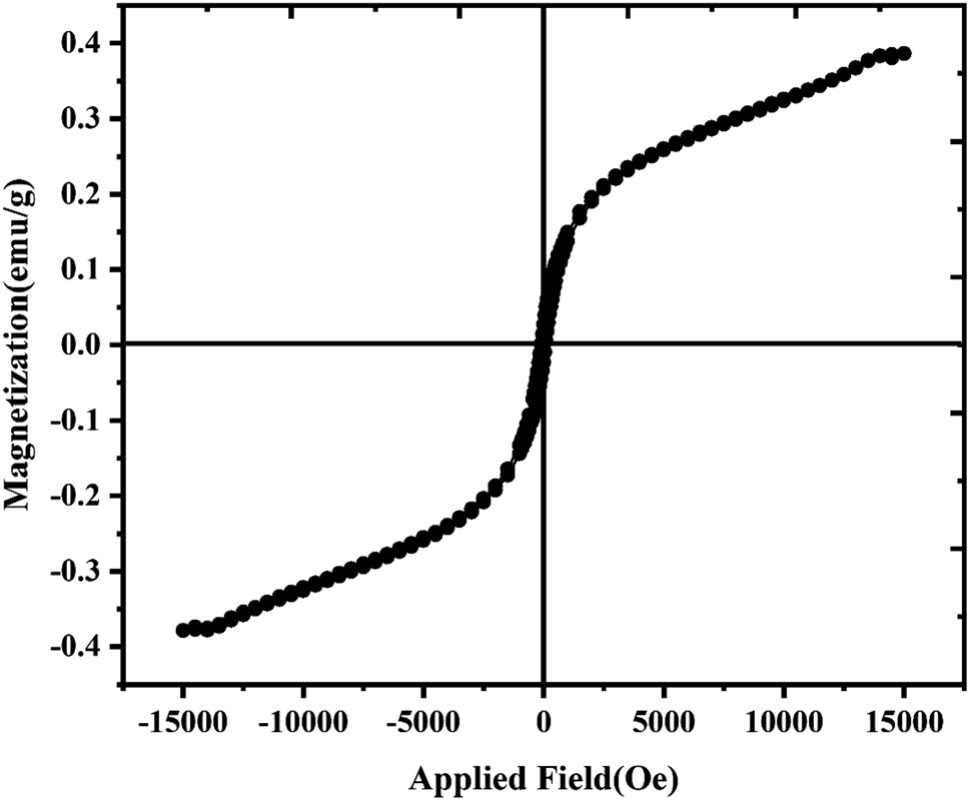
VSM hysteresis of sample 3 at room temperature.

The properties of LiCoO_2_/Fe_3_O_4_/Li_2_B_2_O_4_ nanocomposites in terms of magnetic ability were studied by the VSM technique. The hysteresis loop for sample 3 is displayed in Fig. 8. It can be seen that the plots illustrate a ferromagnetic behavior. Also, the maximum saturation magnetization (Ms) obtained for LiCoO_2_/Fe_3_O_4_/Li_2_B_2_O_4_ nanocomposites with the rod-shaped structure is 0.388 emu.

## 4. Electrochemical hydrogen storage

**Fig. 9:**
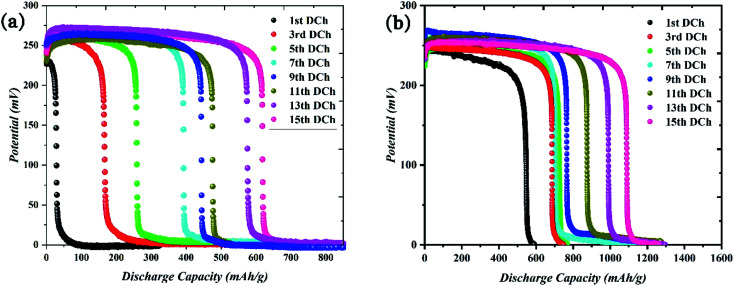
Electrochemical hydrogen storage profiles for 15 cycles. (a) Discharge profiles for bulk (sample 2) and (b) nano rod-shape (sample 3).

Size and morphology are significant factors in energy storage materials. The active materials in the nanoscale can show higher performance than bulk materials due to the surface effects between the electrolyte and active sites in the nanomaterials. The hydrogen sorption capacity for the obtained nanocomposites of LiCoO_2_/Fe_3_O_4_/Li_2_B_2_O_4_ with rod-shaped structures was compared with that of bulk structures. For this purpose, chronopotentiometry tests were conducted in the three-electrode cell (including Pt, Ag/AgCl and working electrodes) in the electrolyte medium of KOH (2 M). The applied current density is constant 1 mA. The discharge capacity for the chosen samples was computed using the *C* = *I*Δ*t*/*m*Δ*V* formula.^19^ The discharge capacity for rod-shaped nanocomposites (sample 3) was 1284 mA h g^−1^ after 15 cycles. However, the bulk sample 2 had a discharge capacity of 694 mA h g^−1^ after 15 cycles (Fig. 9a and b).

**Fig. 10:**
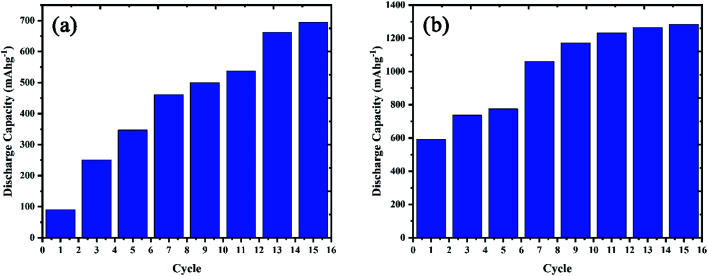
Cycling performance of the fabricated nanocomposites for (a) bulk (sample 2) and (b) nano rod-shape (sample 3).

Diverse mechanisms for the hydrogen storage of metal oxide materials have been proposed, which comprise the existence of layered structures, morphology and physisorption process.^20–22^ The morphology and the shape of electrode materials have an incredible impact on the energy storage proficiency. The borate compounds have open channels and layered structures, which are appropriate for storing the ions and transfer them.^23^ The structure of LiCoO_2_/Fe_3_O_4_/Li_2_B_2_O_4_ nanocomposites assigns facile, rapid and efficacious electron transportation through the electrolyte due to easy access of the electrolyte with the structure of metal oxides. The one-dimensional structures such as wires, rods, or tubes are the most efficient shape for improving the electrochemical proficiency due to the increased surface areas and the shortened ion diffusion paths. The hydrogen storage outcomes figure out a fast surface charge delivery mechanism and higher ion diffusion performance in the electrode materials with the rod-shaped morphology (sample 3), which has a small diameter.^24,25^ The comparative hydrogen sorption study between nano rod-shape and bulk structures of LiCoO_2_/Fe_3_O_4_/Li_2_B_2_O_4_ nanocomposites are shown in Fig. 9. Also, Fig. 10 shows the cycling performance for samples 2 and 3. On the other hand, physical adsorption occurs in the metal oxides during the charge and discharge processes. The electrolyte of 2 M KOH separates into OH^−^ and H^+^ (eqn (1)). Created H^+^ migrates to the working electrode and adsorbs on the surface of the active materials (cathode reaction). The anodic reaction of the charging process occurs in the Pt electrode through an oxidization mechanism. In discharge direction, H_2_ transports from the working electrode under the alkaline circumstance and becomes water again while freeing an electron. The generated H^+^ migrates to the surface of active materials in the fabricated working electrode, which can be adsorbed on the surface of metal oxides. The stored H_2_ in the working electrode should be migrating in the opposite direction when the cell is discharged and converted into water.^26^

1 H_2_O + e^−^ ↔ H + OH^−^

2 LiCoO_2_/Fe_3_O_4_/Li_2_B_2_O_4_ + *x*H_2_O + *x*e^−^ ↔ LiCoO_2_/Fe_3_O_4_/Li_2_B_2_O_4_ − H_*x*_ + *x*OH^−^

3 H + H ↔ H_2_

The Heyrovsky process occurred due to the suitable electrocatalyst activity of the as-synthesized electrode materials. The synergic effects of Tafel (eqn (3)) and Heyrovsky (eqn (2)) processes led to hydrogen storage in the active materials of LiCoO_2_/Fe_3_O_4_/Li_2_B_2_O_4_ nanocomposites. According to the electrochemical outcomes, LiCoO_2_/Fe_3_O_4_/Li_2_B_2_O_4_ nanocomposites have a potential for hydrogen sorption as novel active materials in energy storage fields due to physisorption and rod-shaped morphology.

## 5. Conclusion

In summary, the Pechini-synthesized nanocomposites of LiCoO_2_/Fe_3_O_4_/Li_2_B_2_O_4_ were successfully obtained in the ideal condition using citric acid as a fuel. The hydrogen sorption efficiency is compared with that of the bulk structure and the results show an increase in the discharge capacity from 694 to 1284 from bulk to the nano-sized rod-shaped morphology. Therefore, the magnetic nanocomposites of LiCoO_2_/Fe_3_O_4_/Li_2_B_2_O_4_ as a novel candidate can play a suitable role in the energy storage fields.

## Conflicts of interest

The authors declare that there are no conflicts of interest regarding the publication of this manuscript.

## Supplementary Material
